# Adoption and Use of a Smart Home Connected Care System by Older Adults: Mixed Methods Study

**DOI:** 10.2196/76012

**Published:** 2026-07-15

**Authors:** Laura Pemberton, Michelle Carter, Nikolay Mehandjiev

**Affiliations:** 1 Alliance Manchester Business School University of Manchester Manchester United Kingdom; 2 School of Social Sciences University of Birmingham Dubai United Arab Emirates

**Keywords:** smart home technologies, older adults, technology adoption, UTAUT, Unified Theory of Acceptance and Use of Technology, mixed methods, aging in place, caregiver support, passive monitoring

## Abstract

**Background:**

Smart home technologies are often promoted as a means to support independent living and alleviate pressure on health and care systems. However, adoption among older adults remains low, and little is known about how passive, ambient technologies are experienced over time.

**Objective:**

This study aimed to examine the adoption of a Connected Care System among older adults and their caregivers in a real-world deployment, testing the applicability of the Unified Theory of Acceptance and Use of Technology model and identifying design considerations for future systems.

**Methods:**

A mixed methods study was conducted with 91 older adults who used the Connected Care System for approximately 6 months. Quantitative survey data were analyzed using structural equation modeling to assess predictors of behavior intention. Thematic analysis of open-ended survey responses was used to explore participants’ experiences and perceptions of the system.

**Results:**

Perceived ease of use (β=0.075; *P=*.02) and facilitating conditions (β=0.232; *P*<.001) were significant predictors of behavior intention, while perceived usefulness and social influence were not. Thematic analysis revealed that older adults often conceptualized usefulness in emotional and relational terms, such as peace of mind and caregiver reassurance. Adoption was frequently described as a shared process involving family members. Key barriers included low confidence in setup and reliability concerns, while valued features included passive monitoring, increased independence, and reduced caregiver burden.

**Conclusions:**

The findings suggest that conventional adoption models require adaptation to account for passive, caregiver-mediated smart home systems. Emotional and relational benefits, rather than task efficiency, drive perceived usefulness. Design recommendations are offered to support user-friendly, personalized, and relationally aware smart home technologies that align with the realities of aging in place.

## Introduction

### Background

Smart home technologies are often positioned as part of the solution to the global challenges of population aging, supporting not only older adults but also family members who provide informal care [1]. As the proportion of older adults continues to grow, there is an urgent need for innovative solutions that promote independent living while reducing the emotional and financial pressures on caregivers, who already bear substantial—yet often underreported—burdens [2-4]. Projections indicate that by 2050, one in four people in the United Kingdom will be aged 65 years or older, compared with one in five in 2019 [5]. This demographic shift is expected to place increasing strain on health and social care systems, reinforcing the need to investigate technology-enabled models of care. Within this context, smart home technologies such as the Connected Care System (CCS) developed by a major UK telecommunications company offer a practical example of how digital infrastructure could be leveraged to support aging in place.

Despite their potential, the uptake of smart home technologies remains relatively low among older adults [6-8]. Recent reports show that only 54% of adults over the age of 75 years in the United Kingdom use the internet regularly [9], reflecting broader barriers to digital inclusion. This gap between the growing need for scalable care solutions and the slow adoption of digital technologies underscores the importance of examining how integrated systems, such as the CCS, can address barriers while supporting aging in place. In this study, the CCS consisted of a mobile app used by the informal caregiver, an Amazon Alexa device, and several plug-in motion detector sensors placed in the older adult’s home. The sensors captured patterns of movement and relayed this information through the mobile app, enabling caregivers to receive notifications as the older adult moved about their house. The inclusion of a voice assistant also provided a familiar interface for interaction, while the mobile app acted as the main point of access for caregivers to monitor activity. Unlike professionally monitored telecare systems, the CCS was designed for informal caregiving contexts, where family members received the notifications directly and used them to support independent living. It also supports daily activity tracking, providing caregivers with insights into routines and well-being over time. These functionalities align with ongoing policy and practice goals to extend independent living while addressing the costs associated with formal care provision [10].

However, existing research on smart home technologies for older adults has significant limitations. Much of the evidence comes from laboratory settings or short-term deployments [11], leaving open questions about sustained use in everyday life. For example, the SmartSenior project evaluated a 36-device smart home system over an average of 45 days, finding that acceptance improved once initial technical issues were resolved [12]. Similarly, a 2-year study in a care home context showed that older adults’ attitudes became more positive over time [13]. More recent studies highlight the importance of trust and perceptions of security in influencing adoption [14]. Yet, long-term, real-world evaluations of smart home systems in private households remain rare. This study addresses this gap by examining the adoption and sustained use of the CCS in older adults’ own homes over an extended period, offering novel insights into how these systems function beyond pilot or institutional settings.

This study makes several contributions to the field of technology adoption and smart home system design. First, it evaluates theoretical adoption constructs in the context of passive, ambient smart home technologies, identifying potential gaps where traditional models fail to capture the unique dynamics of older adult users. Second, by integrating qualitative insights, the study refines existing adoption frameworks, offering a more holistic understanding of the interplay between behavior intention, perceived usefulness (PU), social influence, and ease of use [15]. A key practical contribution of this research is the development of 6 design requirements based on empirical findings. These requirements provide actionable insights for technology developers, health care providers, and policymakers seeking to design more accessible, personalized, and user-friendly smart home systems. By bridging the gap between technological potential and real-world adoption, this study supports the broader goal of enabling older adults to maintain independence, improve well-being, and enhance quality of life while aging in place.

### Theoretical Framework

The Unified Theory of Acceptance and Use of Technology (UTAUT) model [15] is often used to predict technology adoption, but it mainly focuses on task-oriented use rather than on in-home or passive use by older adults [16]. While the UTAUT model has been applied to various groups, studies focusing on older adults are scarce [17,18]. When applied, UTAUT has been found sufficient to explain older adults’ technology use [19]. The limited research may be due to the unique needs of older adults, such as physical or cognitive limitations, which can alter the relevance of the model’s core constructs [20,21]. For example, older adults may prioritize ease of use and immediate utility [22] more than younger users. Findings regarding the influence of social factors are also inconsistent [11,23], with some research suggesting a strong influence from family and peers, while other studies find it less significant. Empirical findings also point to mixed results when UTAUT is applied to smart technologies with older adults; for example, one study found that performance expectancy strongly predicted intention to use, but effort expectancy, social influence, and facilitating conditions were not significant, despite being core constructs of the model [24]. However, in other cases, social influence has growing importance due to extra reliance on caregivers [25,26]. These inconsistencies highlight the need for more nuanced research on this demographic. This paper also responds to a call for more thorough research using the UTAUT model that also incorporates qualitative data through a longitudinal study [27].

### Objective

The objective of this study is to investigate the adoption and use of the CCS among older adults during a 6-month real-world deployment. Specifically, we examine the key factors that shape adoption behavior and assess how well-established constructs from the UTAUT explain technology engagement in this context. In addition, this study seeks to identify the perceived benefits and barriers experienced by both older adults and their caregivers. By integrating quantitative measures with qualitative insights, the research provides a nuanced understanding of how smart home technologies are taken up in everyday life and highlights design implications for systems that aim to support independent living and aging in place.

### Research Model and Hypotheses

This study tested the UTAUT model [28] in the context of older adults who had used the CCS for 6 months. The model examines the key determinants influencing older adults’ behavior intention to adopt and use smart home technologies.

Behavior intention refers to an individual’s readiness to perform a specific behavior—in this case, the intention to use smart home technologies [29]. The model includes 4 independent constructs: perceived ease of use (PEOU), PU, social influence, and facilitating conditions. PEOU is defined as the degree to which an individual believes that using a system is free from effort [11]. PU refers to the extent to which a person believes that using the system will enhance their performance or daily living [28]. Together, these constructs capture both the effort and value components of technology acceptance. Social influence reflects the extent to which individuals perceive that people important to them think they should use a technology [28]. This factor is particularly relevant in the context of older adults, whose technology decisions are often shaped by family members and caregivers. Facilitating conditions refer to the degree to which individuals believe that adequate organizational and technical support exists to enable system use [28]. For older adults, these conditions include access to digital infrastructure, such as Wi-Fi, and availability of guidance or assistance during setup [30].

Based on the UTAUT model and the context of this study, the following hypotheses were proposed:

Hypothesis 1: Perceived ease of use positively influences behavior intention to use a smart home system.

Hypothesis 2: Perceived usefulness positively influences behavior intention to use a smart home system.

Hypothesis 3: Social influence positively influences behavior intention to use a smart home system.

Hypothesis 4: Facilitating Conditions positively influence behavior intention to use a smart home system.

## Methods

### Research Design

This study used a longitudinal observational design to examine older adults’ adoption and use of smart home technologies within the context of a real-world deployment. The trial was designed and conducted by a UK telecommunications company, which managed all aspects of recruitment, technology deployment, and data collection. Recruitment was open to households in the United Kingdom that included an older adult receiving informal care from a family member, friend, or neighbor. No other eligibility restrictions were applied. The study commenced in October 2020 but experienced delays due to the COVID-19 pandemic, which influenced the timing and logistics of technology setup in participants’ homes.

A total of 393 participants took part in the trial, with survey completion being entirely voluntary. The smart home technology, the CCS, was installed in participants’ homes with only remote support provided by the telecommunications company. The system included an Amazon Alexa device, motion-detecting smart plugs, and a mobile app that enabled informal caregivers to receive notifications and monitor household activity.

All survey data were anonymized prior to being shared with the researchers. The only information available to the researcher was an overview of participants’ ages, unlinked to any survey responses.

### Ethical Considerations

The data analyzed in this study were collected as part of a trial conducted by a large technology company in the United Kingdom. Ethical approval for the original data collection was obtained through the company’s internal ethics and governance review processes, in accordance with national regulations for industry-led research involving noninvasive technologies. All participants in the original trial provided informed consent to participate. Participation in the survey component was voluntary. This study involved secondary analysis of fully anonymized survey data. No personally identifiable information was available to the research team. In line with UK Health Research Authority guidance on Research Ethics Committee review, research limited to the secondary use of previously collected, non-identifiable information is generally excluded from NHS/HSC REC review where participants are not identifiable to the research team [31]. The authors had permission from the telecommunications company to access and analyze the anonymized dataset.

### Instrument Development

The researcher involved in this study played a pivotal role in designing the survey questionnaire tailored to meet the trial’s specific objectives. Subsequently, the questionnaire was handed over to the organization, the study’s industry partner, for further refinement to ensure alignment with their requirements. Following this collaborative effort in questionnaire design, the researcher’s involvement with the trial concluded, with no direct participation in the trial’s administration. The survey was developed using pre-existing surveys from already recognized models of technology adoption. Constructs selected to be included were primarily based on the UTAUT model [28]. In our instrument development, we opted for the inclusion of open-ended survey questions to provide participants with a platform to articulate their experiences with the CCS. These can also be seen in Multimedia Appendix 1. This design enabled the collection of both quantitative and qualitative data regarding participants’ interactions with the CCS, providing a naturalistic view of technology adoption behavior among older adults.

### Qualitative Analysis

The results reported in this study were derived from an inductive thematic analysis following the 6-stage reflexive approach proposed by Braun and Clarke [32,33]. The analysis used an open-coding strategy, in which participants’ excerpts were first coded line by line and then iteratively grouped into broader categories and overarching themes. This process was conducted using NVivo (version 12, QSR International) software to assist in organizing, coding, and managing the data while enabling the identification of recurring patterns and relationships within the dataset.

The thematic analysis involved the following six stages: (1) becoming familiar with the data through repeated reading and annotation, (2) generating initial codes through open coding of participant excerpts, (3) collating related codes into potential themes, (4) reviewing and refining these themes against the coded data and entire dataset, (5) defining and naming the final set of themes to ensure clarity and distinctiveness, and (6) producing the analytic narrative and selecting illustrative extracts for reporting. Thematic analysis, as a structured and methodical yet flexible approach, was particularly suited to analyzing the open-ended survey responses, allowing for both depth and breadth in interpreting participants’ experiences with the CCS [34].

### Quantitative Analysis

Structural equation modeling (SEM) was used as a social science tool to examine complex relationships among latent constructs and observed variables [35]. In this research, SEM was applied to explore factors influencing attitudes and behaviors towards technology adoption. By combining measurement and structural models, SEM validated instruments and tested causal relationships, making it suitable for small sample sizes like this study’s 91 participants. SEM allowed for efficient analysis by integrating multiple relationships within one model, enhancing reliability despite limited data. The analysis followed standard operational steps: (1) data screening for missing values and outliers; (2) evaluation of the measurement model using Cronbach α and composite reliability (>0.70), average variance extracted (AVE) (>0.50), and discriminant validity via Fornell–Larcker and Heterotrait-Monotrait ratio criteria; and (3) estimation of the structural model using bootstrapping (5000 resamples) to assess path coefficients, *t* values, and *P* values. Model fit was assessed using SRMR, d_ULS, d_G, and NFI indices, which are appropriate for partial least squares SEM. Predictive accuracy was examined through R² values of endogenous constructs. To address limitations, qualitative findings were used to complement SEM results, offering a nuanced view of attitudes towards smart home technologies and balancing potential dataset shortcomings.

### Mixed Methods Analysis

This research used a mixed method approach where quantitative and qualitative research methods were used concurrently, taking an exploratory approach. This research compared the findings of the quantitative and qualitative data collected [15,36]. By comparing and contrasting results from different data sources, triangulation enabled researchers to corroborate findings and uncover nuances that may not have been apparent with either method alone [37].

## Results

### Overview

A total of 91 participants responded to the survey between October 15, 2021, and October 29, 2021. A total of 14% of the participants were younger than 65 years, 16% between the ages of 65 and 75 years, 43% were aged between 75 and 85 years, and finally 27% were older than 85 years across the whole of the study. Additional demographic data were not reported, as only an anonymous dataset was shared.

### Quantitative Analysis

**Figure 1:**
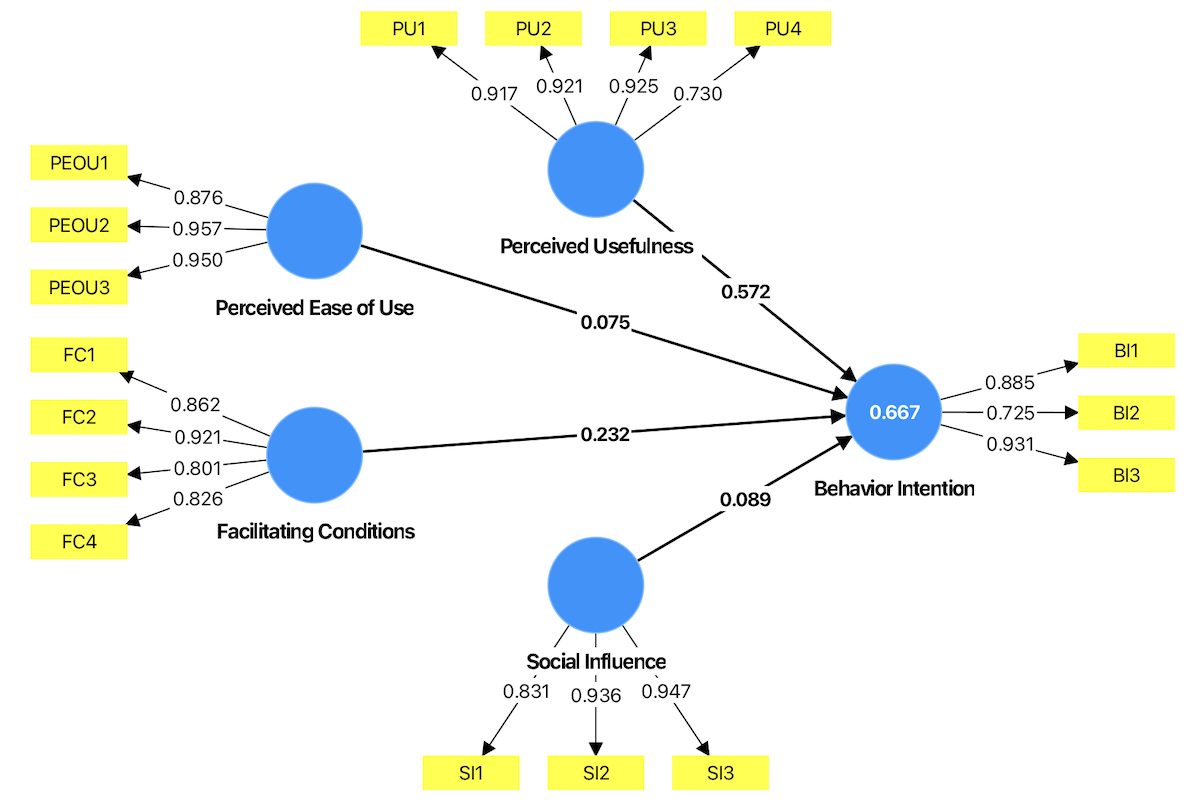
Results of Connected Care System data within the Unified Theory of Acceptance and Use of Technology model of technology adoption. BI: behavior intention; FC: facilitating condition; PEOU: perceived ease of use; PU: perceived usefulness; SI: social influence.

The results of the UTAUT model are shown in Figure 1 and Table 1. PU, PEOU, facilitating conditions, and social influence explained 67% of the variance in behavior intention.

**Table 1:** Unified Theory of Acceptance and Use of Technology model results (*P*<.05 and T>1.96).

	Path coefficients	Sample, mean (SD)	Bootstrapped T value	*P* value	Hypothesis
Hypothesis 1	0.075	0.243 (0.095)	2.441	.02	Supported
Hypothesis 2	0.572	0.071 (0.111)	0.673	.50	Not supported
Hypothesis 3	0.089	0.101 (0.075)	1.186	.24	Not supported
Hypothesis 4	0.232	0.561 (0.099)	5.764	<.001	Supported

When measuring for discriminant validity of the model, we completed 2 separate tests of this. First, by considering the latent variable correlations (Table 2), and second, by considering the Heterotrait-Monotrait ratio ratios (Table 3). The recommended threshold for measuring the latent variable correlations is that all values should be below 0.85 [38]. Values above this can indicate that the constructs are too similar and do not measure distinct enough concepts.

**Table 2:** Unified Theory of Acceptance and Use of Technology latent variable correlations.

	Behavior intention	Facilitating conditions	Perceived ease of use	Perceived usefulness
Facilitating conditions	0.614	—^a^	—	—
Perceived ease of use	0.548	0.697	—	—
Perceived usefulness	0.766	0.502	0.471	—
Social influence	0.507	0.473	0.467	0.477

^a^Not applicable.

**Table 3:** Unified Theory of Acceptance and Use of Technology model discriminant validity (Heterotrait-Monotrait ratio matrix).

	Behavior intention	Facilitating conditions	Perceived ease of use	Perceived usefulness
Facilitating conditions	0.675	—^a^	—	—
Perceived ease of use	0.622	0.768	—	—
Perceived usefulness	0.890	0.520	0.506	—
Social influence	0.570	0.485	0.503	0.524

^a^Not applicable.

The study also tested for construct reliability measures such as Cronbach α and composite reliability (Table 4). The reliability coefficients are all above the recommended threshold of 0.70 [39], indicating satisfactory reliability. AVE assesses the amount of variance captured by the latent variables relative to measurement error. AVE values above 0.50 are generally considered acceptable [40], indicating that constructs explain more variance than measurement error.

**Table 4:** Construct reliability.

	Cronbach α	Composite reliability (rho_a)	Composite reliability (rho_c)	Average variance extracted
Behavior intention	0.808	0.844	0.887	0.726
Facilitating conditions	0.880	0.923	0.915	0.729
Perceived ease of use	0.919	0.940	0.949	0.861
Perceived usefulness	0.897	0.910	0.930	0.769
Social influence	0.891	0.914	0.932	0.821

### Qualitative Results

This results section will describe the most relevant themes found in the thematic analysis, but a full breakdown of themes, including excerpts and frequency counts, is included in Multimedia Appendix 1.

### Acquiring Technology

The largest theme emphasized the significant effort required for technology acquisition (n=46), with participants expressing that they would need substantial support, as one participant explained, “Too much/not capable by myself” (ID74).

Followed by participants’ desire for effortless technology acquisition (n=33), highlighting how important it was that the process of obtaining the CCS felt straightforward. Some participants described the experience as smooth and independent, for example, “It would be pretty straightforward to purchase this product” (ID84). A related theme was the desire to improve knowledge and understanding of the technology (n=37). Participants wanted to make more informed and autonomous decisions. For example, one noted simply, “Understanding of devices” (ID35), pointing to the value of accessible information during the acquisition stage. Transparency of cost information also emerged as a recurring concern (n=30). Participants wanted clarity about setup and ongoing costs to support their decision-making. One participant explained, “I need a simple one price for set up and then one monthly price please, nothing for extras” (ID25). Finally, family advice and support played a key role in acquisition processes (n=23). Many participants relied on family members’ expertise and guidance. As one participant put it, “My nephew as he is very good at explaining how to use the technology in a way I understand” (ID3). In several cases, decisions were made jointly, with either the older adult or the caregiver leading the practical acquisition.

### Attitude to Technology

A prominent theme to emerge was exposure to the latest technology (n=39), which reflected participants’ curiosity and enthusiasm for innovation. Many were eager to explore and keep up with new technological developments, positioning themselves as proactive users. As one participant explained, “To know what new technologies are available and their potential” (ID72). Another described their involvement in testing initiatives: “I wanted to test the new innovation solution from … and wanted to see the latest and be part of the testing. I really enjoyed it” (ID25). This theme highlights how engagement with emerging technology is not solely the domain of younger groups; some older adults also actively seek new experiences.

Closely linked to this was a positive perception of technology (n=34), with participants frequently describing technology as beneficial, particularly for safety and well-being. For instance, one participant stated, “It won’t be going anywhere. It’s been great and I feel much safer” (ID17), while another simply noted “excellent” (ID15). These accounts illustrate the positive value placed on technology as part of everyday life. By contrast, a theme of neutral perception of technology (n=26) was also evident. Here, participants described the technology as “ok,” reflecting moderate satisfaction or indifference rather than enthusiasm or resistance. This suggests a pragmatic stance, where technology is seen as functional but not transformative.

A further theme concerned the role of technology in everyday life (n=24). Participants emphasized the significance of technology in supporting independence and enabling aging in place. For example, one participant noted, “I think it will play an important role in allowing older people to live independent lives in their own homes rather than going into care” (ID22), while another commented, “It will play an effective role in keeping me independent but safe” (ID52). These perspectives position technology as an important, sometimes essential, element of daily living for older adults.

Not all participants, however, saw technology as necessary. The theme of limited need for new technology (n=16) captured participants who stated that they did not perceive a need for the system, suggesting varying levels of perceived necessity based on individual circumstances. A smaller but noteworthy theme was updating current technology systems (n=4), where participants discussed adopting new systems as a way to replace or integrate with their existing devices. Finally, some participants expressed uncertainty about the role of technology (n=15), revealing ambivalence and mixed views. For example, one participant commented, “not too keen” (ID10), while another remarked, “It’s not really impinged on my life. It’s more for the kids” (ID29). These accounts reflect uncertainty about both the relevance and impact of the technology.

### Older Adults’ Perspective

A central theme concerns mobility decline (n=53), which emerged as one of the most significant challenges for participants. Many described increasing difficulties in moving around their homes, particularly for essential activities such as eating or using the bathroom. As one participant put it, “Getting around the house for food, toilet, etc” (ID52). Another noted, “I need a walking aid as I’m not good on my feet and can't walk far” (ID64). This theme underscores the ways in which declining mobility shapes daily routines and heightens vulnerability. Several participants expressed concerns about falls (n=15), including fears of collapsing alone at home, as one participant put it, “fear of failing” (ID29). Linked to this were concerns around memory decline (n=14), with participants describing difficulties remembering tasks, appointments, or daily routines. For example, one participant said, “My memory isn’t so good so it’s not so easy for me to remember what I’m supposed to be doing and when” (ID22). Closely related to this was the theme of health decline (n=25), which captured broader concerns about deteriorating physical capacities, including sight and general health. For example, one participant noted, “Further deterioration in my sight” (ID36), reflecting a heightened awareness of age-related change and its implications for independence.

Alongside these physical changes, participants also described challenges with solo tasks and chore difficulty (n=22), highlighting the impact of functional decline on everyday activities. For instance, one participant referred to a “decline in day to day functioning” (ID28), while another noted difficulties with “carrying things around house safely” (ID39). Emotional and social dimensions were also prominent. The theme of loneliness and isolation (n=18) revealed feelings of spending much of the day alone and a desire for companionship. One participant described, “feel lonely at times” (ID41), and another emphasized, “The ability to be able to have human interaction and company” (ID20). Similarly, mental health decline (n=9) captured experiences of boredom, demotivation, and low mood, as in the comment, “Boredom, not seeing anybody” (ID80).

A smaller theme captured older adults’ reluctance to rely on assistive systems (n=13). Some participants articulated a preference for maintaining autonomy, for example: “but I want to be able to do things myself and ask for help if needed not rely on it” (ID25). This theme points to the tension between independence and technological support. Another theme, growing need for emergency response (n=8), reflected participants’ recognition of the benefits of continuous monitoring and rapid assistance. As one participant explained, “Need more monitoring / access to immediate help” (ID70). These accounts illustrate the desire for reassurance that support is available in emergencies. Finally, the theme of reduced frequency of welfare checks (n=7) highlighted the perceived value of technology in supporting independent living while easing caregiver burden. One participant commented, “Less calls from relatives checking on me as they can see from the app I’m moving” (ID43). This was seen to benefit both older adults, who experienced fewer interruptions, and caregivers, who could monitor well-being remotely.

### System Benefits

Participants described multiple benefits of the CCS, particularly in enhancing well-being and quality of life. A prominent theme was the older person’s peace of mind (n=45), reflecting reassurance from knowing that assistance is available in case of emergencies. One participant explained, “I am always seeking ways of giving me more peace of mind that if something happens to me, I won’t be alone” (ID68), while another noted, “have some relief knowing that my family are able to be notified if something happens” (ID86). Similarly, the theme of caregiver’s peace of mind (n=26) highlighted how the system alleviates family concerns: “it stops my family worrying about me, knowing where I am or if I am alright” (ID25).

The CCS also supported enhanced independence (n=26), empowering older adults to live autonomously at home. Participants emphasized this benefit, as one stated, “Being able to live alone longer” (ID45), and another added, “I want to stay in my own home and live independently and these solutions will help me do that” (ID64). Complementing this was the theme of feelings of connectedness (n=13), illustrating how the system fosters familial bonds: “secure I know my daughter is looking after me” (ID10) and “Knowing my family are feeling more connected to me” (ID7).

Practical utility was also emphasized through the theme of usefulness (n=20). Participants described how the system supported daily activities and safety, for instance: “it is like the front door lock, I can’t do without, even if I think I am safe!!” (ID25). Relatedly, caregiver support (n=16) reflected how the CCS facilitates informal caregiving by providing monitoring and communication: “It’s just used by my family to watch over me” (ID64). Improved communication (n=15) further emphasized connectivity and prompt response in emergencies, as one participant noted, “Confidence there is a way of contacting people and being monitored in case I have issues” (ID28). Several participants valued discreet monitoring and devices (n=34 and n=11, respectively), highlighting unobtrusive design and passive oversight: “I know that I can be seen to be active without feeling like I’m being watched” (ID60) and “I think the devices all look very smart and fit into my home nicely” (ID68). Finally, ease of use (n=5) and reminders (n=4) were seen as additional benefits, with participants describing the system as simple and supportive: “Very simple” (ID70) and “Little reminders are great” (ID3).

### System Issues

The most prevalent theme concerned the reliability of the emergency support system (n=49). Participants consistently expressed worries about their ability to contact someone in case of emergencies, highlighting the need for a dependable and responsive system. One participant stated, “Making sure I can contact someone if I need to” (ID40), while another emphasized, “Fear of not being able to contact someone in the event of emergency” (ID6). These concerns underline that the perceived trustworthiness of emergency support is central to adoption and continued use.

Closely related was the theme of requiring additional and properly functioning devices (n=20). Participants highlighted the importance of reliable call buttons and integrated devices to enhance system effectiveness. As one participant noted, “Our call button never worked and we would definitely want one of those” (ID74), while another commented, “More but needs greater integration with other devices” (ID78). This theme reflects broader concerns about technology reliability and the need for well-functioning, complementary devices. The theme of assistance with setup and maintenance (n=9) captured participants’ need for practical support when installing or maintaining the system. Many reported struggling with new technology or complex setup procedures. For example, one participant explained, “Simple advice as I do not understand new technology” (ID61), and another stated, “I would not understand it or be able to do it without the help of others” (ID37). These accounts highlight that effective user support is critical for ensuring proper use.

Participants also emphasized dependence on additional services or devices (n=7), reflecting a desire for customizable solutions that meet individual needs and preferences. One participant noted, “Video system is likely to be more useful” (ID6), while another stated, “Also if additional types of device become available, they would be considered” (ID74). This theme underscores the value of flexibility in system design to accommodate varying requirements. Smaller themes included financial aid (n=14), with participants recognizing that affordability may influence adoption, and adjustment periods required to trust technology (n=3), exemplified by a participant remarking, “It took a bit of time for me to trust it wasn’t watching everything I do” (ID60). These reflect practical and psychological considerations that can affect engagement with the system.

## Discussion

### Principal Findings

This study examined how older adults perceive and engage with the CCS, a passive smart home technology designed to support well-being through ambient monitoring. Using a mixed methods approach, we identified several insights that extend existing theoretical models and carry implications for the design, implementation, and policy contexts of care technologies. Notably, our findings suggest that UTAUT [28] requires adaptation when applied to ambient systems. For older adults who engage with the system indirectly, the boundary between PU and behavior intention becomes less meaningful. Consistent with prior research [16,41-44], PU and social influence did not reach statistical significance, highlighting the complexity of measuring technology adoption in this demographic. This finding suggests that PU may require reconceptualization in the context of passive or ambient smart home technologies. For older adults, usefulness may not be derived from direct task performance or system efficiency—as traditionally defined in UTAUT—but from indirect, emotional, and relational benefits, such as reassurance, safety, and reduced caregiver burden. Redefining PU to incorporate these affective and social dimensions may therefore provide a more accurate representation of technology value in aging in place contexts.

A key factor shaping these findings is the passive nature of the CCS. Unlike technologies used actively on a day-to-day basis, participants rarely interacted directly with the system or the data it collected. Instead, benefits were realized indirectly through caregivers or family members, who could monitor the older adult’s well-being without interrupting their daily routines. Consequently, participants’ perceptions of usefulness were primarily emotional rather than functional [14], encompassing peace of mind, reassurance, and ease of access to safety information. As one participant noted, the comfort provided by the system is valuable even if it is not actively used. This underscores the importance of evaluating technology not only in terms of functional outcomes but also in terms of emotional and relational value.

The study also highlights the critical role of social influence and caregiver mediation in technology adoption. Although quantitative analysis found no significant link between social influence and behavior intention, qualitative findings emphasized that family and caregivers play a central role in adoption and ongoing use. This reflects prior research in health settings that integrates social, emotional, and contextual factors into technology acceptance models [45] and suggests that adoption should be conceptualized as a relational process involving shared decision-making and co-dependency within household care arrangements. These dynamics raise broader concerns about digital equity, particularly in how older adults rely on others to access and benefit from smart home technologies.

It is important to acknowledge the potential impact of the small sample size (n=91) on the statistical power of the SEM and the generalizability of the findings. While SEM provides a robust framework for evaluating complex relationships, smaller sample sizes can increase the risk of Type II errors, potentially obscuring significant effects and limiting the reliability of path estimates. In this study, the limited sample may have contributed to the nonsignificant relationships observed between PU, social influence, and behavior intention. Furthermore, the participant pool reflects a specific demographic context—older adults engaged with a passive smart home system—which may not capture the full diversity of older populations in different care settings or cultural contexts. These considerations highlight that, while the findings provide meaningful insights into the relational and emotional dimensions of technology adoption, caution is warranted in extrapolating the results beyond this sample. Future research should aim to replicate these analyses with larger and more diverse cohorts to confirm the robustness of the observed patterns.

Furthermore, this study contributes to methodological advancements in technology adoption research by demonstrating the value of mixed methods approaches. While UTAUT has been widely applied using survey-based models [46], our research underscores the necessity of integrating qualitative thematic analysis to contextualize adoption behaviors [45]. The discrepancies between quantitative and qualitative findings, particularly regarding PU and social influence, highlight the limitations of relying solely on numerical measures to understand technology adoption in aging populations. By combining statistical modeling with in-depth user perspectives, this study provides a richer, more nuanced understanding of technology adoption, reinforcing the importance of mixed methods research in studying complex user interactions with emerging technologies [23,47].

Ultimately, this research contributes to ongoing debates about how emerging technologies are transforming domestic life and care infrastructures. By integrating qualitative thematic analysis with a widely used adoption model, the study provides a socially embedded account of technology uptake—reflecting the realities of aging, caregiving, and digital participation. The findings highlight the importance of designing technologies not only for individual users but also for the networks and systems that support them, with consideration for emotional reassurance, relational dynamics, and passive engagement.

### Comparison With Prior Work

The joint table developed from the mixed method analysis can be seen in Multimedia Appendix 1 [36]. The table presents the qualitative and quantitative findings alongside the construct relationships found in our CC model. The final column of the table presents the meta inference, and the distinction of each inference as being in discordance, expansion or confirmation [48].

### Behavior Intention

The quantitative findings of this study provide strong evidence that behavior intention is a significant driver of technology adoption among older adults, as indicated by the high explanatory power in our UTAUT model. This suggests that older adults are not only receptive to smart home technologies like the CCS but also actively recognize their potential to enhance quality of life, increase independence, and provide peace of mind. These findings align with previous studies demonstrating that when technology addresses key psychological and emotional needs, older adults are more willing to adopt and use it [11,49]. The qualitative data further support this conclusion, revealing a strong enthusiasm for continued use of the CCS. Participants particularly valued the peace of mind, security, and independence it provided, not just for themselves but also for their caregivers. However, while the overall attitude toward adoption was positive, qualitative insights also highlight key challenges and barriers that must be addressed to ensure sustained, reliable, and comfortable usage. These include concerns about reliability, usability, and the role of caregivers in facilitating technology adoption. This suggests that technology adoption among older adults is not simply a matter of willingness—rather, it is shaped by contextual factors, such as how well the system integrates into their existing support networks and whether it is perceived as empowering rather than burdensome.

### Perceived Ease of Use

Our quantitative findings confirm that PEOU is a significant predictor of behavior intention. This aligns with the technology acceptance model, reinforcing the well-established importance of usability in driving technology adoption among older adults [29]. High outer loadings for PEOU indicators suggest that participants consistently found the CCS to be intuitive and learnable, supporting the need for simplified and user-friendly interfaces. The qualitative findings, however, provide additional depth to this perspective. While participants acknowledged that the system was relatively easy to use, they also emphasized the importance of customizability and flexibility. Many older adults desired the ability to mix and match features based on their individual needs, which were largely influenced by health status, mobility, and caregiving arrangements [50]. This suggests that ease of use is not a one-size-fits-all construct—rather, it should be viewed as a dynamic, individualized factor that varies based on personal circumstances and support systems. A notable insight from the qualitative data is that older adults often rely on caregivers or family members for technology setup, troubleshooting, and maintenance. This highlights an important but underexplored dimension of PEOU: the role of the caregiver in facilitating ease of use. Some participants saw the caregiver as the primary user, while they themselves merely benefited from the system’s presence. This raises a critical question: how does ease of use function in systems with shared users, particularly when the technology benefits both the caregiver and the older adult?

### Perceived Usefulness

The qualitative data strongly emphasized the usefulness of the CCS, with participants highlighting key benefits such as peace of mind, safety, and independence. These benefits were not just functional but also emotional, reinforcing findings that technology for older adults often plays a psychosocial role beyond simple task efficiency [11,49]. However, the quantitative results indicate that PU did not significantly predict behavior intention. This discrepancy suggests that the traditional conceptualization of PU in technology adoption models may not fully capture its meaning for older adults. Specifically, usefulness in this context extends beyond functionality—it encompasses emotional security, social connectedness, and caregiver support. Furthermore, the qualitative findings suggest that customizability plays a key role in perceptions of usefulness. Older adults highly valued the ability to tailor the system to their specific needs, which were highly individualized based on health, mobility, and social support structures [50].

### Social Influence

The relationship between social influence and behavior intention was the weakest in our model, a finding that aligns with previous studies questioning the role of social influence in older adults’ technology adoption [23,47]. This suggests that broader social networks (eg, friends, community) have minimal impact on adoption decisions, while immediate family and caregivers play a much more significant role [49,51]. The qualitative findings reinforce this idea, revealing that older adults primarily rely on caregivers and family members for support, setup, and decision-making. Interestingly, participants often saw the caregiver as the true decision-maker, which may explain the weak correlation between social influence and behavior intention—older adults do not see the choice as their own, but rather as a necessity dictated by their caregivers. This suggests a reconceptualization of social influence is needed in models of technology adoption for older adults, particularly when technology is used collaboratively.

### Facilitating Conditions

The quantitative findings confirm that facilitating conditions are a strong predictor of behavior intention, reinforcing existing literature that emphasizes the importance of external support in technology adoption [52]. However, our qualitative findings reveal a more complex picture of facilitating conditions. The moderate positive correlation between facilitating conditions and PEOU (0.697) suggests that the availability of resources and support contributes to users’ perceptions of how easy the system is to use. This underscores the importance of providing adequate support structures, clear instructions, and accessible help resources to enhance the PEOU of smart home systems for older adults. Particularly in the case of older adults, who may be starting their adoption process from a point of less knowledge than younger users of smart home systems. A key theme emerging from the data is that older adults perceive facilitating conditions as external rather than internal. Many participants felt that they did not need personal knowledge of the system, as they relied on family members to provide expertise and manage technology-related tasks. This suggests that the traditional framing of facilitating conditions in UTAUT may need revision—rather than measuring an individual’s perceived ability to use technology, future models should account for interdependent technology adoption, where one party (the caregiver) facilitates access for another (the older adult). Additionally, the qualitative data highlight the importance of knowledge acquisition—older adults expressed a need for better education and guidance on how to use CCS effectively. This aligns with prior research indicating that older adults require ongoing education and training to improve confidence in new technology [52].

### Implications for Design and Practice

The findings from this study inform a set of 6 interrelated design requirements that enhance the adoption, usability, and effectiveness of smart home technologies such as the CCS for older adults and their caregivers. Together, these requirements highlight the importance of designing systems that are not only technically reliable and easy to use but also emotionally supportive, socially connected, and adaptable to users’ evolving needs. They address key adoption factors, including behavior intention, facilitating conditions, social influence, PU, and ease of use. Table 5 summarizes how each construct informed specific design requirements, linking empirical insights to actionable design guidance.

**Table 5:** Design requirements showing the meta inference made across each of the constructs, the system adaptation required to fit in the smart home context, and the requirements.

UTAUT^a^ construct	Meta-inference	Requirement based on adaptation
Perceived usefulness	Older adults and caregivers value peace of mind, security, and independence provided by CCS^b^. High behavior intention indicates a strong interest in adoption.	RQ1: Provide clear and tangible benefits emphasizing safety, independence, and emotional reassurance.
Perceived ease of use	Older adults often lack confidence and require structured onboarding to navigate the system effectively.	RQ2: Implement an intuitive, guided onboarding process with tutorials and voice navigation. RQ3: Develop a minimalist, senior-friendly UI with large fonts, voice controls, and simple navigation.
Social influence	Immediate family plays a crucial role in adoption, highlighting the need for real-time shared insights into user well-being.	RQ4: Include a “Shared Dashboard” for real-time well-being monitoring by family members.
Facilitating conditions	Concerns about reliability affect perceived usefulness; trust-building measures can increase confidence in the system.	RQ5: Emphasize trust-building features (fail-safe mechanisms, backup options, transparent logs); provide a dual-interface option with “Essential Mode” and “Full Feature Mode”, allowing users to customize their experience based on their comfort level.
Behavior intention	Older adults have varying comfort levels with technology; dual-interface options allow for gradual adaptation and personalized interaction.	RQ6: Provide dual-interface options to allow gradual adaptation and personalized interaction, supporting both mediated and autonomous use.

^a^UTAUT: Unified Theory of Acceptance and Use of Technology.

^b^CCS: Connected Care System.

A central design priority is to ensure that smart home technologies deliver clear and tangible benefits (RQ1). The findings show that both older adults and caregivers highly value peace of mind, safety, and independence, making these factors essential in encouraging adoption. To reinforce behavior intention, systems should communicate emotional and practical benefits early and consistently, for example, through onboarding materials, real-life success stories, and user testimonials that illustrate improvements in daily life and well-being. Integrating real-time feedback, alerts, and status updates can further build confidence and demonstrate reliability, reassuring users that the system is functioning as intended [11].

For many older adults, low digital confidence remains a significant barrier to adoption. A structured onboarding process that includes step-by-step tutorials, voice-assisted navigation, and simplified instructions can help users build comfort and familiarity (RQ2). Importantly, training should not target the older adult alone. Because family members and caregivers often play a central role in setup and troubleshooting, implementing a “Family Support Mode” would allow them to provide remote assistance when needed. To maintain inclusivity, designers should also offer multiple access pathways, such as an on-demand helpline, chatbot, or low-tech phone-based interfaces, ensuring that support is available for users who struggle with app-based interactions [52].

Ease of use also depends on minimizing cognitive load and supporting habitual use. A senior-friendly interface with large fonts, voice commands, and clear navigation can make the system more accessible (RQ3). Compatibility with smart assistants such as Alexa or Google Home would reduce interaction complexity and align with technologies many users already know. Personalized automation features—such as preset routines and scheduled alerts—can further reduce manual adjustments and promote effortless engagement. These design strategies together enhance usability and sustain engagement over time, reinforcing the link between ease of use and adoption [23].

Equally important is recognizing that adoption and use are shared experiences rather than solitary ones. A “Shared Dashboard” can enable caregivers to remotely monitor well-being while preserving older adults’ autonomy (RQ4). Multiuser support and customizable permission settings allow older adults to determine what data are visible and to whom, maintaining control and privacy within supportive relationships. These features echo evidence that family involvement—rather than peer influence—is the strongest predictor of adoption among older adults [50,51]. To further enhance trust, fail-safe mechanisms, transparent data logs, and customizable alerts (eg, emergency notifications, wellness check-ins, or reminders) should be integrated. A non-intrusive “Check-in” feature can also allow caregivers to confirm safety in a way that is supportive rather than surveillant [49].

Beyond interpersonal support, the system itself must feel dependable and seamlessly embedded in daily life. Smart home interfaces should be transparent, intuitive, and designed for users with differing levels of digital experience (RQ5). Because many participants preferred minimal engagement, passive monitoring functions should operate quietly in the background, providing meaningful support without creating a sense of constant observation or disruption [45]. This principle extends beyond product design to service delivery, offering guidance for housing providers, health and social care organizations, and policymakers seeking to integrate smart home systems into aging in place strategies [53].

Finally, long-term adoption depends on flexibility and personalization. Older adults’ needs and comfort levels change over time, meaning that a single interface or configuration is unlikely to suit all users indefinitely. A dual-interface option, allowing users to switch between a simplified Essential Mode and a more advanced Full Feature Mode, supports gradual learning and adaptation (RQ6). Customization features—such as adjustable settings, notification preferences, and tailored prompts—ensure that technology remains user-centric and empowering. Such personalization not only promotes sustained engagement but also helps mitigate feelings of surveillance or dependency, reinforcing autonomy and dignity in everyday use [30,50]. In this way, personalization and emotional reassurance become mutually reinforcing elements of inclusive smart-home design. This form of personalization enhances usability while helping to reduce perceptions of surveillance or disruption, especially in multiuser households [54].

Taken together, these design implications emphasize that effective smart home technologies for aging populations must integrate emotional reassurance, shared use, and adaptability into their core functionality—not as optional features but as central principles. Doing so will enable systems like the CCS to support aging in place while strengthening trust, confidence, and connectedness between older adults and their caregivers [55].

### Limitations and Future Research

Building on the contributions of this study, future researchers should rethink traditional approaches to technology adoption in aging populations by incorporating training interventions and structured support systems that better address older adults’ needs. This study highlights the importance of customization and personalization, suggesting that future research should move beyond generic usability considerations and instead investigate which specific customization features are most effective in promoting long-term engagement with smart home and health care technologies.

Additionally, this study challenges the conventional social influence construct in technology adoption models. Future researchers should redefine social influence by considering how shared-use systems operate when one user group (eg, caregivers) has more agency in adoption decisions than the other (older adults). Researchers should explore family dynamics in adoption processes, shifting focus from broad social influence on immediate social networks, particularly how family members shape adoption and continued use. Future studies should explore how caregiver involvement modifies traditional adoption pathways, particularly in health, caregiving, and assistive technologies [52]. A key limitation of this study is that data were collected solely from older adults, without corresponding input from their caregivers. As a result, the findings reflect older adults’ perceptions of caregiver involvement rather than caregivers’ own experiences and perspectives.

Furthermore, this work calls for a broader conceptualization of PU in aging populations. Future adaptations of UTAUT should refine the PU construct to better capture indirect, relational, and emotional benefits, rather than relying solely on task efficiency-based measures [11,49]. Last, researchers should integrate care models with technology acceptance frameworks, recognizing that smart home technologies are often collaborative rather than individualistic, requiring a more nuanced understanding of adoption behaviors across multiple stakeholders.

The methodology used in this study was subject to several noteworthy limitations, which significantly impacted the results and constrained the interpretation of the data. The lack of control over participants’ access to technology introduced variability in their technological usage throughout the experiment. This may have confounded the results, particularly affecting the PEOU construct. By not having an in-depth understanding of the user’s existing experience and self-efficacy with computer use, the results and their interpretation have assumed minimal experience and knowledge regarding computer skills. Despite these limitations, the study’s strength lies in its use of real participants who used a real system for a minimum of 6 months. This real-world usage provides valuable practical insights, reflected in the strong relationship between facilitating conditions and behavior intentions. However, the methodological constraints may have led to an incomplete picture of the adoption process, particularly in understanding the temporal aspects of adoption and the evolving perceptions of usefulness over time.

### Conclusions

This study sought to examine the adoption and use of the CCS among older adults over a 6-month period, addressing a critical research gap in understanding how this population interacts with smart home technologies in real-world settings. By using both quantitative and qualitative methods, the study offers a more nuanced perspective on the factors influencing the adoption of these technologies. The research contributes to the existing literature by testing the applicability of preexisting adoption constructs, particularly those from the UTAUT model, while also revealing insights that may challenge or extend these frameworks when applied to older adults. Findings indicate that PU in passive smart home technologies must be redefined to include emotional and relational benefits, rather than focusing solely on task efficiency. The role of caregivers as facilitators in adoption decisions emerged as crucial, necessitating a more relational approach to social influence.

Furthermore, personalization and adaptability were identified as key factors in sustained engagement. These insights have practical implications for system design, emphasizing the need for user-friendly, customizable, and caregiver-supported technologies. Future research should further explore caregiver involvement, shared-use adoption models, and long-term engagement strategies to ensure that smart home technologies effectively support aging in place, balancing independence, usability, and support structures.

## Appendix

Multimedia Appendix 1 Quantitative and qualitative survey instruments, thematic analysis results with code frequencies and participant excerpts, mixed methods meta-inferences, and resulting design requirements.

## Abbreviations

AVE: average variance extracted
CCS: Connected Care System
PEOU: perceived ease of use
PU: perceived usefulness
SEM: Structural Equation Modeling
UTAUT: Unified Theory of Acceptance and Use of Technology
