# Supplementary material for: Adoption and Use of a Smart Home Connected Care System by Older Adults: Mixed Methods Study
Source: JMIR Aging. 2026 Jul 15;9:e76012. doi: 10.2196/76012 (PMC13420526; doi:10.2196/76012)
Supplement: Multimedia Appendix 1 [file aging_v9i1e76012_app1.docx]

**Multimedia Appendix 1**

Appendix A – Survey Instrument

| Construct | Item | Question | Adapted From |
| --- | --- | --- | --- |
| Behaviour Intention | BI2 | I expect to use the technology in my home daily | Pal, Funilkul, Vanijja, et al. (2018b) |
|  | BI1 | I intend to get additional smart home devices |  |
|  | BI3 | I plan to continue to use the technology. | Venkatesh et al. (2003b) |
| Perceived Ease of Use | PEOU2 | I can use the smart devices on my own | Davis et al. (1989) |
|  | PEOU1 | it is easy for me to use the technology | Davis et al. (1989) |
|  | PEOU3 | using the devices does not require any amount of special effort or energy. | Davis et al. (1989) |
| Perceived Usefulness | PU2 | Using this technology improved the performance of my daily tasks | Davis et al. (1989) |
|  | PU3 | I can accomplish my daily tasks more quickly with this technology | Davis et al. (1989) |
|  | PU1 | there is an improvement to my Quality of Life with this technology | Davis et al. (1989) |
|  | PU4 | I am satisfied that the assisted living technology will provide me with support in an emergency |  |
| Facilitating Conditions | FC3 | I have the time to use Connected Care | (Venkatesh, 2012) |
|  | FC4 | I have the knowledge necessary to use the technology | (Venkatesh, 2012) |
|  | FC2 | adopting a smart home system is entirely in my control | (Venkatesh, 2012) |
|  | FC1 | I am confident using the smart home technology in my daily life. | (Venkatesh, 2012) |
| Social Influence | SI3 | AAL was recommended to me by people whose opinion I trust | (Venkatesh, 2012) |
|  | SI1 | People who are important to me think that I should use the smart home technology | (Venkatesh, 2012) |
|  | SI2 | People who influence my behaviour think I should use the smart home technology | (Venkatesh, 2012) |

Appendix B - Qualitative Data Questions

What are the main challenges you face when you are alone?

What part of your life/wellbeing do you think is most likely to change as a result of Connected Care?

What situation in your life would push you to seek out assistive technology solutions?

What do you need to support you in purchasing this technology?

How much effort would it be for you to purchase and use additional devices after the trial?

Do you need any additional skills or expertise to be able to purchase and use this technology in the future?

What were you hoping to get from being part of the Connected Care trial?

Will you be looking to add any additional devices once the trial has ended?

How do you perceive the technology that has been new to you in the trial?

How frequently do you use the system?

In the future what role do you expect this type of technology to play in your everyday life?

Appendix C - Themes

Acquiring Technology

| Theme | Excerpt | Count |
| --- | --- | --- |
| Effort for tech acquisition | “Too much / not capable by myself” – ID74  “reasonable - needs to be made easy” – ID85  “A lot, I would need help” – ID43 | 46 |
| Knowledge improvement | “Understanding of devices” – ID35  “knowledge that it will help resolve my problems” – ID28 | 37 |
| Effortless tech acquisition | Participants stated no help required – multiple  “easy” – ID79  “It would be pretty straight forward to purchase this product.” – ID84 | 33 |
| Transparency of cost information | “It will depend how much it all costs especially with the heating bills going up so much,” – ID60  “Not too much effort ... might need to re-budget, depends on other household rising costs versus benefit” – ID70  “visibility of price points” – ID11  “i need a simple one price for set up and then one monthly price please, nothing for extras” – ID25  “A clear indication of options and costs” – ID65 | 30 |
| Family advice and support | “My nephew as he is very good at explaining how to use the technology in a way I understand.” – ID3  “I don't understand these things but the family know what's best and what I can use” – ID59 | 23 |

Attitude to technology

| Theme | Excerpt | Count |
| --- | --- | --- |
| Exposure to latest technology | “To know what new technologies are available and their potential.” – ID72  “experience of new technology” – ID67  “i wanted to test the new innovation solution from … and wanted to see the latest and be part of the testing. i really enjoyed it” – ID25 | 39 |
| Positive perception of Tech | “excellent” – ID15  “It won’t be going anywhere. It’s been great and I feel much safer.” – ID17  “I love tech so anything new” – ID15 | 34 |
| Neutral perception of tech | Participants described that they perceived the technology as “ok” | 26 |
| Role of tech in everyday life | “I think it will play an important role in allowing older people to live independent lives in their own homes rather than going into care” – ID22  “Necessary”- ID34  “It will play an effective role in keeping me independent but safe.” – id52 | 24 |
| Limited need for new technology | Participants described not needing the technology system. | 16 |
| Uncertainty about tech role | “not too keen” – ID10  “mixed, some elements works great and others not helped at all” – ID11  “It’s not reallly impinged on my life . It’s more for the kids.” – ID29 | 15 |
| Updating Current Tech Systems | “To be able to ditch the alarm monitoring system I have” – ID40  “a vital part if the whole solution works and can be integrated with other devices (Alexa, webcam etc)” – ID11 | 4 |

Older adults’ perspective

| Theme | Excerpt | Count |
| --- | --- | --- |
| Mobility decline | “Getting around the house for food, toilet, etc”– ID52  “I need a walking aid as I'm not good on my feet and can't walk far” – ID64 | 53 |
| Health decline | “Further deterioration in my sight” – ID36  “If my health were to deteriorate more” – ID26 | 25 |
| Solo tasks & chore difficulty | “decline in day to day functioning” – ID28  “Carrying things around house safely” – ID39 | 22 |
| Loneliness and Isolation | “feel lonely at times” – ID41  “Loneliness, someone to talk to” – ID70  “The ability to be able to have human interaction and company” – ID20 | 18 |
| concerns about falls | “, fear of failing” – ID29  “having a health scare in the house such as significant fall or passing out for a period of time”-ID86 | 15 |
| Memory decline | “remembering tasks/appointments” – ID67  “My memory isn’t so good so it’s not so easy for me to remember what I’m supposed to be doing and when.” – id22  “Remembering to turn off heating, lights or hot water.” – ID3 | 14 |
| older user will not use/rely on system | “but i want to be able to do things myself and ask for hlep if needed not rely on it.” – ID25 | 13 |
| No lifestyle change | Participants explained that they saw no change to their life as a result of using this technology | 11 |
| Mental health decline | “Boredom, not seeing anybody,” – ID80  “Possible issues with depression - tech solution to ask how i am feeling routinely and report possible issues to family/carers” – ID74  “Demotivated” – ID37 | 9 |
| Growing need for emergency response | “Need more monitoring / access to immediate help” – id70  “Being able to get in touch with someone if this happened in the future would push be to look into more solutions.” – id63 | 8 |
| Reduced frequency of welfare checks | “Less calls from relatives checking on me as they can see from the app I'm moving” – ID43  “I wont get as many anxious calls from friends and relatives who check up on me! The regular catch up calls are fine!” – ID65 | 7 |

System benefits:

| Theme | Excerpt | Count |
| --- | --- | --- |
| Older person's peace of mind | “I am always seeking ways of giving me more peace of mind that if something happens to me I wont be alone.” – ID68  “have some relief knowing that my family are able to be notified if something happens” – ID86 | 45 |
| Discreet monitoring | “I know that I can be seen to be active without feeling like I'm being watched” – id60  “Yes everything will be easier to passively monitor me.” – id29 | 34 |
| Safety & security perception | “Knowing that alerts will be sent to my loved ones if no movement is detected makes me feel safer in my home.” – ID22  “Safety and security and knowing im being looked after” – ID22 | 34 |
| Caregiver's peace of mind | “it stops my familiy worrying about me, knowing where i am or if i am alrgiht” – id25  “Peace of mind for my carer that I'm ok” – ID18 | 26 |
| Enhanced Independence | “Being able to live alone longer” – ID45  “I want to stay in my own home and live independently and these solutions will help me do that” – id64 | 26 |
| Usefulness | “Very little use, video system is likely to be more useful” – ID6  “it is like the front door lock, i can't do without, even if i think i am safe!!” – ID25  “I think it’s good and beneficial to me” – ID22 | 20 |
| Caregiver support | “It's just used by my family to watch over me.”- ID64  “hoping to make checking up easier” – ID39  “To please my close family who wanted to be more connected and keep an eye out for me.” -ID7 | 16 |
| Improved Communication | “Confidence there is a way of contacting people and being monitored in case I have issues” – ID28 | 15 |
| Feelings of connectedness | “secure I know my daughter is looking after me” – ID10  “Knowing my family are feeling more connected to me: - ID7 | 13 |
| Discreet devices | “I think the devices all look very smart and fit into my home nicely,” – ID68  “Wonderful and I've forgotten the sensors are there as it is very unobtrusive” – ID64  “was worried it would have wires everywhere. That wasn't the case and it has been invisible.” – ID60 | 11 |
| ease of use | “Very simple” – ID70  “Excellent and trouble free” – ID8  “As long as the technology is simple an intuitive to use I’m happy” – ID22 | 5 |
| reminders | “Little reminders are great” – ID3  “It would be good to act as a reminder for doing the tasks that I may forget to do as I get older,” – ID20 | 4 |

System Issues:

| Theme | Excerpt | Count |
| --- | --- | --- |
| Reliability of emergency support system | “Making sure I can contact some one if I need to” – id40  “fear of failing and not able to alert anyone” – ID29  “Fear of not being able o contact someone in the event of emergency” – ID6 | 49 |
| Requires additional & working devices | “Our call button never worked and we would definitely want one of those.” – ID74  “More but needs greater integration with other devices” – ID78  “Probably not, but perhaps an alert button.” – ID7  “i do like the trends on the app, they should come on the tablet and compare to last week or last month, tablet is so much larger than the phone and eaiser to read, so if it can come on the tablet so much better.” – ID25 | 20 |
| Financial Aid | "I suppose the obvious thing is just available spare money"-ID20  “I do not have much in the way of pension or benefits so it would be a decision I would have to make and balance out with other things.” – ID64 | 14 |
| Assistance with setup and maintenance | “Simple advice as I do not understand new technology” – ID61  “their help to setup any kit (can't do anything like that myself) and for them to provide user "support/maintenance"” – id74  “I would not understand it or be able to do it without the help of others” – ID37  “Yes as the original setup was quite hard” – ID57  “I honestly wouldn't know how to set it up, I would need assistance.” – ID73 | 9 |
| Dependence on additional services/devices (customisability of service) | “video system is likely to be more useful” – ID6  “That depends on what is available a” – ID28  “It depends on whether I feel the device will be of benefit to me. “ – ID22  “Also if additional types of device become available they would be considered.” – ID74 | 7 |
| adjustment period required to trust technology | “It took a bit of time for me to trust it wasn't watching everything I do” – ID60 | 3 |

Appendix D – Meta inferences

| Concept for Comparison | Qual | Quant | Mixed Method Inference |
| --- | --- | --- | --- |
| Behavioural Intention | The qualitative data revealed a strong focus on the benefits of the Connected Care System (CCS), especially the peace of mind, enhanced independence, and security it provided to older adults and caregivers. Participants valued the system for its role in improving their quality of life. | Behavioural Intention explained 67% of the variance in technology adoption (R² = 0.667). High scores indicate strong interest in adopting the technology, with users showing a general inclination to use it. | Confirmation: The strong behavioural intention shown in the quantitative data aligns with the positive themes from the qualitative analysis. Older adults and their caregivers value the CCS for its emotional and practical benefits, especially the psychological comfort it brings, which likely drives their intention to adopt the technology. |
| Facilitating Conditions | Participants frequently emphasized their need for family support and improved knowledge to effectively acquire and use the technology. They expressed concerns about lacking the confidence to manage the system independently, highlighting the cognitive and emotional reliance on others for support. | Hypothesis supported (Path coefficient = 0.232, p < 0.001). Facilitating Conditions were a strong predictor of Behavioural Intention. | Expansion: The qualitative results from this study can be used to expand on the findings of the quantitative results. The path coefficient between these two variables can be explained by the qualitative data that largely suggests that the older adults in the sample do not have enough knowledge to feel competent at using the connected care system. However, these quantitative and findings can be contextualised by the qualitative data. Perhaps this is because the older adult considers the family member as being all the facilitating conditions that they need in order to acquire such technology. |
| Social Influence | The qualitative findings suggest that family support played a pivotal role in technology adoption. Participants heavily relied on their immediate social network (family and caregivers) to help them acquire and use the CCS. However, broader social networks (friends, community) had little to no influence. | Hypothesis not supported (Path coefficient = 0.089, p = 0.236). Social Influence did not significantly predict Behavioural Intention. | Discordance: Path coefficient is very weak. Suggesting that social influence does NOT influence a person’s attitude towards the technology. These quantitative findings are in discordance with the qualitative findings, that appear to suggest the social influence and benefits of using the technology are quite important to the older user. This could be suggestive of the fact that a broader social network has very little influence on an older person’s use of smart home technology (perhaps because of social stigma), but that the immediate social network such as family play a crucial role in supporting the adoption of such technologies.  The older user relies on the advice and support of family to be able to choose and acquire the technology, so much so that the family can be described as the facilitator of the technology. As well as desiring to use the technology to better support their caregiver in providing care for them as well as providing them with peace of mind. |
| Perceived Usefulness | The qualitative data strongly emphasized the emotional benefits of the CCS, including peace of mind, safety, and independence. Participants noted that these benefits often came from knowing that they and their caregivers were supported, rather than from performing tasks more efficiently.  Qualitative data also highlighted lack of trust in reliability of the CCS. This can have influenced the participants perception of how useful the system is. | Hypothesis not supported (Path coefficient = 0.572, p = 0.501). Perceived Usefulness was not a statistically significant predictor of Behavioural Intention. | Discordance: The qualitative data shows a strong emphasis on the usefulness of the Connected Care system, with themes like "Older person's peace of mind," "Safety & security perception," and "Enhanced Independence" being frequently mentioned. However, the quantitative results show that perceived usefulness was not a statistically significant predictor of behavioural intention (path coefficient 0.572, T-statistic 0.673, p = 0.501). This discrepancy suggests that while users recognize the system's benefits, these perceptions may not directly translate into intention to use.  The qualitative themes reveal that usefulness is a complex construct encompassing various aspects such as peace of mind, safety, independence, and caregiver support. This multifaceted nature may not be fully captured in the quantitative measure of perceived usefulness, potentially explaining the discrepancy between qualitative and quantitative findings. These benefits are largely regarding interpersonal relationships and not directly from a task being completed more effectively/efficiently with a piece of technology.  Themes like "Feelings of connectedness" and "Improved Communication" highlight emotional and psychological benefits that may contribute to behavioural intention in ways not directly measured by the perceived usefulness construct in the quantitative model. |
| Perceived Ease of Use | Participants repeatedly highlighted the effort required to use the system and the need for support in managing the technology. The cognitive effort involved in learning new systems, along with the need for customization and ease of use, were critical for older adults. | Hypothesis supported (Path coefficient = 0.075, p = 0.015). Perceived Ease of Use significantly predicted Behavioural Intention. | **Confirmation:** The alignment between qualitative and quantitative findings suggests that ease of use is a crucial factor in technology adoption for this demographic. The strong emphasis on effort and support needs in qualitative data reinforces the importance of designing intuitive, accessible, and customizable systems for older users. The significant path coefficient further highlights that reducing cognitive effort and improving usability are key drivers of adoption. |

Appendix E: Requirements

| Requirement Number | Requirement | Meta Inference |
| --- | --- | --- |
| 1 | Provide clear and tangible benefits emphasizing safety, independence, and emotional reassurance. | Older adults and caregivers value peace of mind, security, and independence provided by CCS. High behavioural intention indicates strong interest in adoption. |
| 2 | Implement an intuitive, guided onboarding process with tutorials and voice navigation. | Older adults often lack confidence and require structured onboarding to navigate the system effectively. |
| 3 | Include a 'Shared Dashboard' for real-time well-being monitoring by family members. | Immediate family plays a crucial role in adoption, highlighting the need for real-time shared insights into user well-being. |
| 4 | Emphasize trust-building features (fail-safe mechanisms, backup options, transparent logs). | Concerns about reliability affect perceived usefulness; trust-building measures can increase confidence in the system. |
| 5 | Develop a minimalist, senior-friendly UI with large fonts, voice controls, and simple navigation. | Cognitive load is a barrier; simplifying UI with clear navigation enhances usability. |
| 6 | \| Provide a dual-interface option with 'Essential Mode' and 'Full Feature Mode', allowing users to customize their experience based on their comfort level. \| \| --- \| | \| Older adults have varying comfort levels with technology; dual-interface options allow for gradual adaptation and personalized interaction. \| \| --- \| |
